# Biomechanical adaptations for burrowing in the incisor enamel microstructure of Geomyidae and Heteromyidae (Rodentia: Geomyoidea)

**DOI:** 10.1002/ece3.7765

**Published:** 2021-06-16

**Authors:** Daniela C. Kalthoff, Thomas Mörs

**Affiliations:** ^1^ Department of Zoology Swedish Museum of Natural History Stockholm Sweden; ^2^ Department of Palaeobiology Swedish Museum of Natural History Stockholm Sweden

**Keywords:** biomechanics, enamel microstructure, geomorpha, heliscomyidae, North America, rodent incisors

## Abstract

The enamel microstructure of fossil and extant Geomyoidea (Geomyidae, Heteromyidae) lower incisors incorporates three‐ or two‐layered schmelzmusters with uniserial, transverse Hunter‐Schreger bands having parallel and perpendicular or exclusively perpendicular oriented interprismatic matrix. Phylogenetically, these schmelzmusters are regarded as moderately (enamel type 2) to highly derived (enamel type 3). Our analysis detected a zone of modified radial enamel close to the enamel–dentine junction. Modified radial enamel shows a strong phylogenetic signal within the clade Geomorpha as it is restricted to fossil and extant Geomyoidea and absent in Heliscomyidae, Florentiamyidae, and Eomyidae. This character dates back to at least the early Oligocene (early Arikareean, 29 Ma), where it occurs in entoptychine gophers. We contend that this specialized incisor enamel architecture developed as a biomechanical adaptation to regular burrowing activities including chisel‐tooth digging and a fiber‐rich diet and was probably present in the common ancestor of the clade. We regard the occurrence of modified radial enamel in lower incisors of scratch‐digging Geomyidae and Heteromyidae as the retention of a plesiomorphic character that is selectively neutral. The shared occurrence of modified radial enamel is a strong, genetically anchored argument for the close phylogenetic relationship of Geomyidae and Heteromyidae on the dental microstructure level.

## Graphical abstract

Incisors of fossil and extant Geomyidae (pocket gophers) and Heteromyidae (pocket mice, spiny pocket mice, kangaroo mice, kangaroo rats) show enamel with built‐in failure prevention related to chisel‐tooth digging. This distinctive microstructure character strengthens the joint phylogenetic origin of these North American rodents because it is absent in other (extinct) clades within Geomorpha (Eomyidae, Heliscomyidae, Florentiamyidae).

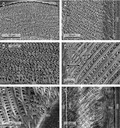

## 1 INTRODUCTION

Although extant New World Geomyidae (pocket gophers) and Heteromyidae (pocket mice, spiny pocket mice, kangaroo mice, kangaroo rats) vary considerably in gross morphology and behavior (small scansorial forms, bipedal hoppers, and robust burrowers), they are united in the clade Geomyoidea based on cranial and dental morphology, soft anatomy, fossil records, molecular data, and biogeography (Flynn et al., 2008; Hafner, 1993). Their most characteristic features are the unique large, fur‐lined cheek pouches external to the oral cavity, from which the word "pocket" in their name originates (Morton et al., 1980). More recent molecular and morphologic studies agree on a close phylogenetic relationship of Geomyidae and Heteromyidae; however, they found conflicting results suggesting extant members of the Heteromyidae to be monophyletic (Hafner et al., 2007; Upham et al., 2019; Wahlert, 1985) or paraphyletic with Geomyidae nested within (Asher et al., 2019; DeBry, 2003; Fabre et al., 2012; Upham et al., 2019).

Modern geomyoids are endemic to North, Central, and northern South America, and their fossil record dates back to the early Oligocene (Orellan, 33 Ma) (Korth, 1993). Geomyoids and related geomorphs are well represented in Cenozoic faunas across North America, and in the Oligocene and Miocene epochs, they were diverse and radiated into several extinct lineages, such as Heliscomyidae, Florentiamyidae, entoptychine Geomyidae, and Mioheteromyinae, particularly in the Great Plains and adjacent mountain regions (Asher et al., 2019; Flynn et al., 2008). Most authors suggest Geomyidae and Heteromyidae as sister group to the extinct basal geomorphs Heliscomyidae and Florentiamyidae and phylogenetically related to the extinct Eomyidae (Asher et al., 2019; Fahlbusch, 1985; Flynn, 2008; Flynn et al., 2008; Jiménez‐Hidalgo et al., 2018; Korth, 1994; McKenna & Bell, 1997).

**FIGURE 1:**
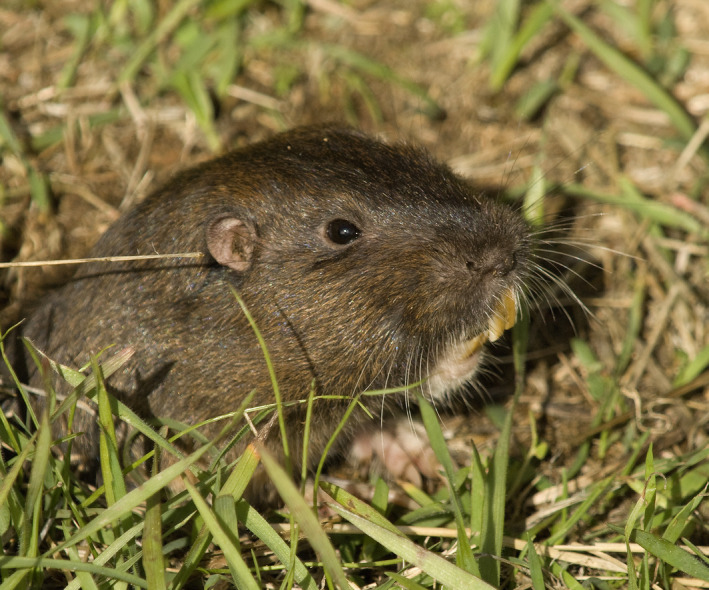
The chisel‐tooth digging Botta's Pocket Gopher *Thomomys bottae* (Eydoux and P. Gervais, 1836) emerging from its tunnel. Photo credit: Chuck Abbe, Nine Sisters Photography, Wikimedia Commons (CC BY 2.0)

Geomyidae (pocket gophers) are small‐ to medium‐bodied rodents with anatomies highly adapted to a subterranean‐burrowing (fossorial) lifestyle in open habitats. They possess a wedge‐shaped, massive skull with a broad, forward‐sloping occipital surface, heavy muscle attachments, and protruding lower incisors that are used as chisels for tunneling by representatives of Thomomyini (MS Hafner, 2016; Wahlert, 1985) (Figure 1). The lips enclose the posterior side of the incisors, preventing the ingestion of sediment while tooth‐digging. Strong and long claws on the front legs are used for shoveling, especially in the scratch‐digging Geomyini (MS Hafner, 2016). The earliest fossil record of Geomyidae is entoptychine gophers from the early Oligocene (Arikareean) (Flynn et al., 2008). Geomyid findings from the early Arikareean (early Oligocene) in the Pacific Northwest and Northern Rocky Mountains (Calede & Rasmussen, 2020; Samuels & Hopkins, 2017) point to a broad distribution of geomyids early in their history. Full exploitation of the subterranean niche in geomyids dates back to at least the early Oligocene (early Arikareean, Ar1), when the entoptychine gopher *Gregorymys veloxikua* created tooth‐excavated foraging burrows in southern Mexico (Jiménez‐Hidalgo et al., 2018; Ortiz‐Caballero et al., 2020). Nevertheless, there likely was more paleoecological diversity in early members of the clade (other than *Gregorymys*) with some taxa possibly semi‐fossorial (not subterranean), others more fossorial or, in contrast, adapted to a terrestrial lifestyle (Calede et al., 2019). It should be mentioned that Asher et al. (2019) placed *Gregorymys* outside Geomyoidea, a view that our study does not support.

Heteromyidae (pocket mice, spiny pocket mice, kangaroo mice, kangaroo rats) are small‐bodied rodents (DJ Hafner, 2016). Some representatives, especially dipodomyines, have adaptations to a hopping or saltatorial locomotion expressed by elongate hind limbs, convergent with Old World gerbils (Voorhies, 1975). Like geomyids, they live in self‐constructed burrows with variable complexity depending on the species. However, heteromyids are anatomically not as strongly adapted to a subterranean lifestyle as their fossorial relatives. Skull morphology varies considerably across heteromyids but the cranial bone is thin and papery and not robust like geomyid skulls. The skulls of some taxa, especially dipodomyines and perognathines, have inflated auditory bullae (Flynn et al., 2008; Korth & Samuels, 2015; Wahlert, 1985). The oldest Heteromyidae are known from the early Oligocene (Orellan, 33 Ma) of North America, and there is fossil evidence for saltatorial locomotion in the Miocene dipodomyines *Cupidinimus* (Hermingfordian to Hemphillian, 18–5 Ma) and *Eodipodomys* (Clarendonian, 10 Ma) and for inflated auditory bullae in the early Miocene (late Arikareean to early Hemingfordian, 23–18 Ma) perognathine *Bursagnathus* (Korth & Samuels, 2015; Samuels & Hopkins, 2017; Voorhies, 1975; Wood, 1935).

One of the basal geomorph sister groups to Geomyoidea is the extinct Heliscomyidae, a family of very small‐bodied rodents ranging from the middle Eocene to early Miocene (Duchesnian to Hemingfordian, 40–16 Ma) of North America (Flynn et al., 2008; Korth, 1994; Korth et al., 1991; McKenna & Bell, 1997). The origin of heliscomyids is unclear; Geomyidae and Eomyidae have been considered their closest relatives (Engesser, 1999; Fahlbusch, 1985; Korth, 1994). A recent study (Asher et al., 2019) suggests Heliscomyidae as ancestral to all extant Geomyoidea.

Lower incisor schmelzmuster and incisor morphology of North American geomorph rodents have not been studied in detail previously. Only a few random fossil and extant taxa were analyzed by Wahlert (1968) and Kalthoff (2000).

Our motivation for this study is a serendipitous discovery of a conspicuous microstructure (i.e., modified radial enamel) in the lower incisors of *Thomomys*, *Chaetodipus*, and *Dipodomys*. Here, we intend to (a) describe the schmelzmuster and lower incisor morphology in a representative sample of fossil and extant Geomyoidea (Geomyidae, Heteromyidae), (b) document the occurrence of modified radial enamel, (c) discuss its assumed biomechanical and higher level phylogenetic implications, and (d) compare these enamel microstructure results with examples in basal geomorphs (Heliscomyidae).

## 2 MATERIALS AND METHODS

Enamel microstructure analysis is a powerful tool for answering systematic–phylogenetic questions at the genus and higher taxonomic levels. Enamel formation is controlled by genetic and epigenetic factors; as a consequence, few samples of each taxonomic subgroup are sufficient to characterize its main schmelzmuster features.

Here, we describe the schmelzmuster of lower incisors of ten species of Geomyidae, ten species of Heteromyidae, and one or two species of Heliscomyidae. The sample from geomyoids covers taxa from the late Oligocene/Early Miocene (Arikareean) to Recent, the two heliscomyid samples come from the late Eocene (Chadronian) and early Oligocene (Orellan) (Appendix S1).

In geomyines, the enamel microstructure is similar in upper and lower incisors. However, as in many rodent clades, upper and lower incisors in Dipodomyinae and Perognathinae have different schmelzmuster with the lower incisor being more apomorphic (Kalthoff, 2000). Only lower incisors were available for sectioning for Entoptychinae, Mioheteromyinae, Heteromyinae, and Heliscomyidae. For reasons of comparability, we chose lower incisors for this study. Schmelzmuster type denotations follow Kalthoff (2000), and character polarity for microstructure characters follows Martin (1999). Enamel thickness categories are as follows: less than 70 µm relate to thin; between 71 and 90 µm relate to moderate; greater than 90 µm relate to thick; and greater than 140 µm relate to very thick.

Preparation for enamel microstructure analysis follows Kalthoff (2000) and Koenigswald and Mörs (2001). Transverse and longitudinal sections were studied and documented using a scanning electron microscope (SEM) (Quanta FEG 650, located at the Swedish Museum of Natural History in Stockholm), at acceleration voltages of 15–20 kV and magnifications of x 30 to × 5,000. All measurements are given in µm and were carried out on transverse sections.

Institutional abbreviations. **AMNH**, American Museum of Natural History, New York, USA. **F:AM**, Frick collection of the AMNH; **IGPB**, Institute of Geosciences, Section Palaeontology, University of Bonn, Germany. **KOE**, enamel collection established by Wighart von Koenigswald, housed in the IGPB. **MVZ**, Museum of Vertebrate Zoology, Berkeley, USA. **NRM**, Swedish Museum of Natural History, Stockholm, Sweden. **TMM**, Texas Memorial Museum, Austin, USA. **UM**, University of Michigan, Ann Arbor, USA. **USNM**, National Museum of Natural History, Smithsonian Institution, Washington D.C., USA. **UK**, University of Kansas Natural History Museum, Lawrence (KS), USA. **ZSHD,** Zoologische Sammlung Heidelberg, Germany.

Anatomical Abbreviations. **EDJ**, enamel–dentine junction; **HSB**, Hunter‐Schreger band(s); **IPI**, inner portio interna; **IPM**, interprismatic matrix; **MRE**, modified radial enamel; **OPI**, outer portio interna; **OES**, outer enamel surface; **PE**, portio externa; **PI**, portio interna; **PLEX**, prismless layer.

## 3 RESULTS

All taxa feature uniserial Hunter‐Schreger bands (HSB). Table 1 summarizes the results and measurements; Appendix S2 gives the raw result data per specimen.

**TABLE 1:** Summary table of measurement ranges, schmelzmuster type, stratigraphic age, and taxonomic information for the analyzed taxa

Taxa	No of species	Schmelzmuster type	Thickness of E (µm)	PI or IPI (µm)	%	OPI (µm) if present	%	PE (µm)	%	Modified radial enamel
GEOMYIDAE										
Geomyinae (Pleistocene/Holocene/extant)	4	2/2a	94–142	65–96	50–69	7–15	6–14	22–55	23–39	YES
Geomyinae (Mio/Pliocene)	3	2/2a	90–97	50–55	52–61	6–16	7–16	27–31	30–32	YES
Entoptychinae	3	2/2a	94–120	43–65	46–57	11–19	12–16	33–40	30–42	YES
HETEROMYIDAE										
Dipodomyinae (extant)	1	3	120–133	85–94	70–71	0	0	35–39	29–30	YES
Dipodomyinae (Miocene)	2	2	94–120	59–76	63	12	10–13	23–32	24–27	YES
Heteromyinae	1	3a	144	116	80	0	0	28	20	YES
Perognathinae (Pleistocene/Holocene/extant)	3	3	96–110	73–85	75–77	0	0	23–25	23–25	YES
Perognathinae (Pliocene)	2	3	90–114	60–75	66–67	0	0	30–39	33–34	YES
Mioheteromyinae	1	2	161	97	60	17	11	47	29	YES
HELISCOMYIDAE										
Heliscomyidae (Eocene/Oligocene)	1–2	2a	64–66	33–35	51–53	10	15–16	21	32–33	NO

Abbreviations: E, enamel; IPI, inner portio interna; OPI, outer portio interna; PE, portio externa; PI, portio interna.

### 3.1 Geomorpha

#### 3.1.1 Geomyoidea

##### Geomyidae: Entoptychinae

###### *Gregorymys* cf. *curtus* (Figures 2f and 3h), Early Miocene: latest Arikareean, KOE 3256

This taxon has a three‐layered schmelzmuster (schmelzmuster type 2) with continuous transversely oriented HSB showing a steep inclination. In the IPI, HSB decussate at high acute angles and the IPM is perpendicular to the prism long axes. In the thin OPI, the IPM turns into a very low angled to parallel direction in respect to the prisms. At the EDJ is a 2–3 prism thick zone, in which the HSB decussate at a low angle forced by the plate‐like IPM in between them. The PE is made up of radial enamel. The incisor cross section is triangular‐shaped with labially flat enamel.

**FIGURE 2:**
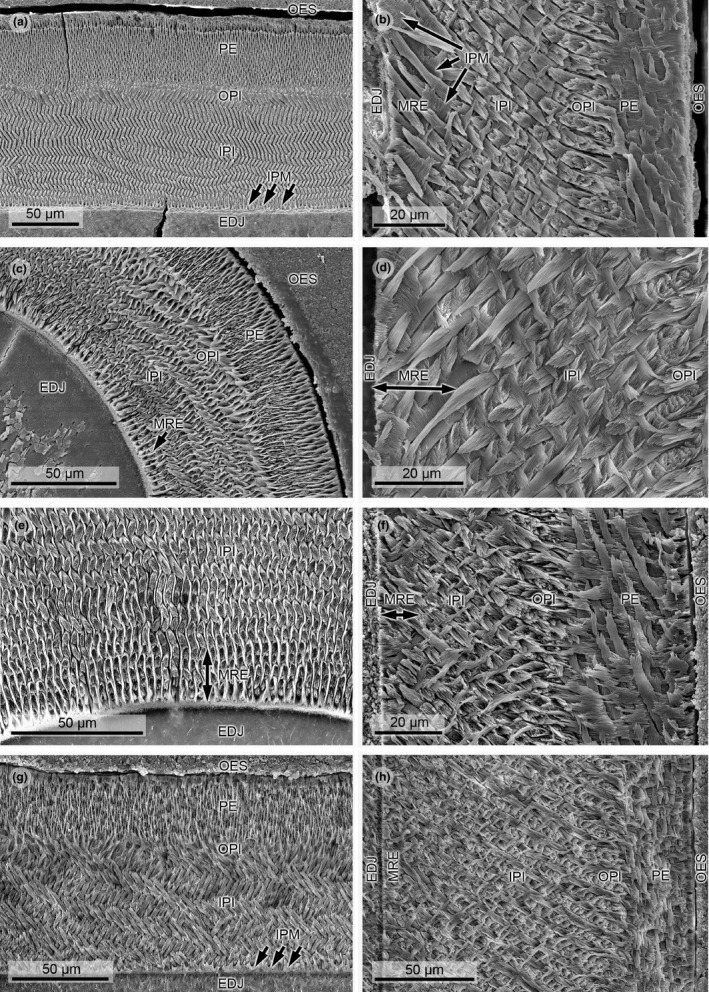
Scanning electron micrographs of lower incisor enamel microstructure in Geomyidae. (a) *Thomomys talpoides*, transverse section, KOE 650. Arrows point to thickened IPM. (b) *Geomys quinni*, longitudinal section, KOE 3248. Arrows point to plate‐like IPM. (c) *Geomys* sp., transverse section at bend toward lateral, KOE 3511. Modified radial enamel extends over two to three prism rows. (d) *Thomomys bottae*, detail of longitudinal section, KOE 3283. Modified radial enamel with plate‐like IPM is well expressed. (e) *Cratogeomys castanops*, transverse section, KOE 3284. Obvious modified radial enamel with plate‐like IPM, extending over about five prism rows. (f) *Gregorymys* cf. *curtus*, longitudinal section, KOE 3256. Modified radial enamel is already present in this entoptychine gopher genus, which is the first to appear in the early Oligocene in North America. (g) *Entoptychus* sp., transverse section, KOE 3244. Arrows point to thickened IPM. (h) *Pleurolicus* sp., longitudinal section, KOE 3257. Modified radial enamel is rather thin in this genus. Abbreviations: EDJ, enamel–dentine junction; IPI, inner portio interna; IPM, interprismatic matrix; MRE, modified radial enamel; OPI, outer portio interna; OES, outer enamel surface; PE, portio externa

**FIGURE 3:**
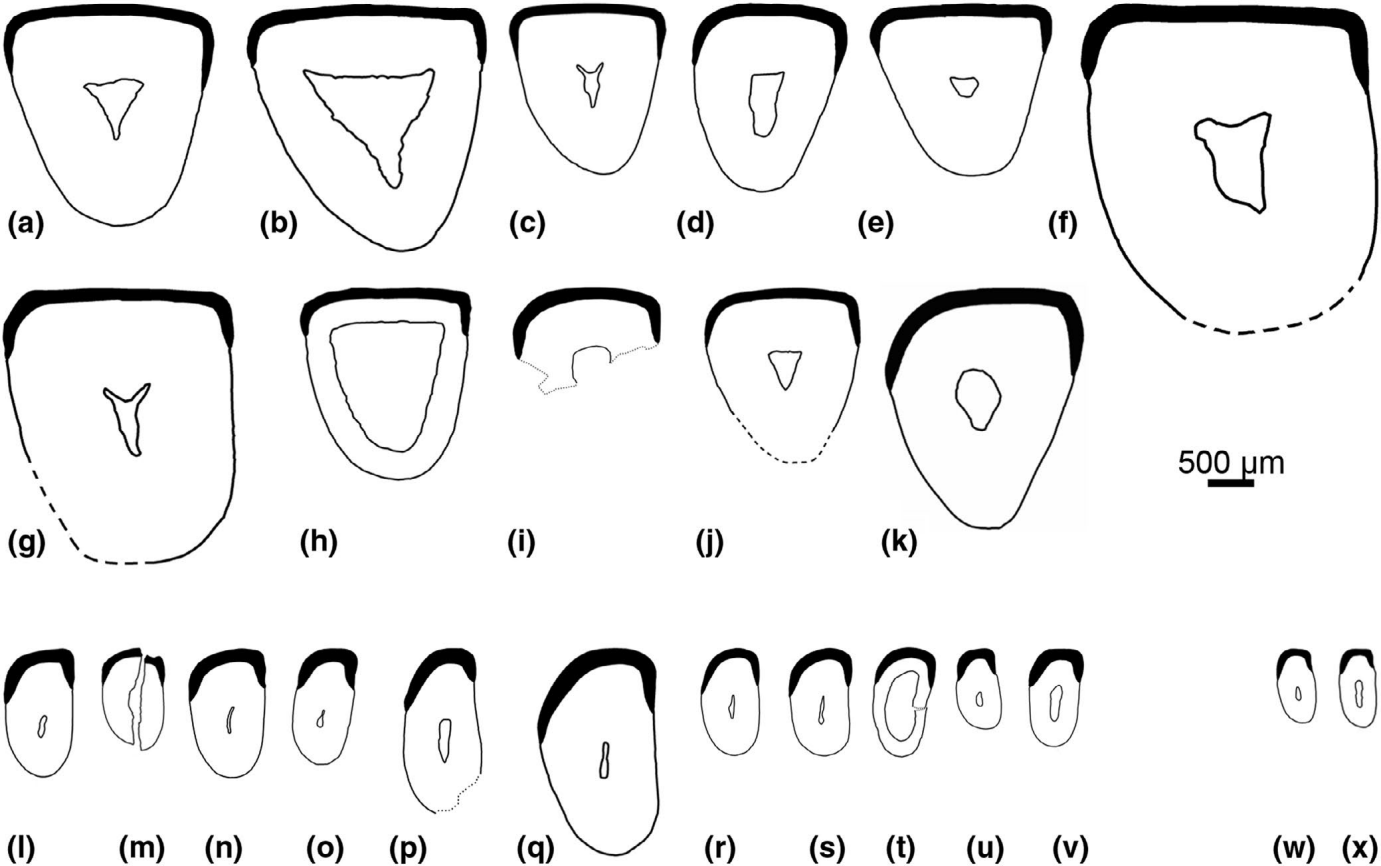
Lower incisor cross sections of the analyzed Geomyidae (a–k), Heteromyidae (l–v), and Heliscomyidae (w–x). All cross sections are shown as left side and drawn to scale. (a) *Pliogeomys buisi*, KOE 3500. (b) cf. *Geomys quinni*, KOE 3248. (c) *Geomys* sp., KOE 3511. (d and e) *Geomys bursarius*, KOE 3275, 3279. (f) *Cratogeomys castanops*, KOE 3284. (g) *Entoptychus* sp., KOE 3244. (h) *Gregorymys* cf. *curtus*, KOE 3256. (i) *Pleurolicus* sp., KOE 3257. (j) *Thomomys bottae*, KOE 3283. (k) *Thomomys talpoides*, KOE 650. (l) *Cupidinimus* cf. *cuyamensis*, KOE 3496. (m) *Cupidinimus nebraskensis*, KOE 3259. (n and o) *Dipodomys ordii*, KOE 1011, 1601. (p) *Heteromys anomalus*, KOE 3431. (q) *Schizodontomys sulcidens*, KOE 3270. (r) *Perognathus* *mclaughlini*, KOE 3505. (s) *Perognathus* *rexroadensis*, KOE 3507. (t) *Perognathus* *bibalii*, KOE 3252. (u) *Perognathus* *merriami*, KOE 3274. (v) *Chaetodipus penicillatus*, KOE 1602. (w) *Heliscomys* sp., KOE 3466. (x) *Heliscomys vetus*, KOE 3528

###### *Entoptychus* sp. (Figures 2g and 3g), Early Miocene: late Arikareean, KOE 3244

*Entoptychus* sp. has a three‐layered schmelzmuster with transversely oriented HSB showing moderate to steep inclination; HSB are diagonally oriented at the mesial and lateral ends of the enamel (schmelzmuster type 2a). The enamel thickens somewhat at the bend to mesial and lateral. The PI is made up of a thick IPI, in which HSB decussate at a generally high angle except for a 1–2 prism thin zone near the EDJ where the decussation angle is low because of thick plate‐like IPM. In the equally thin OPI (1–2 prisms thick), IPM runs parallel to the prism long axes. The PE has radial enamel. The incisor cross section is subtriangular in shape with a rounded dentine body and labially flat enamel.

###### *Pleurolicus* sp. (Figures 2h and 3i), Late Oligocene/Early Miocene: Arikareean, KOE 3257

*Pleurolicus* sp. shows a three‐layered schmelzmuster (schmelzmuster type 2) with transversal HSB having a moderate to steep inclination. At the bend to mesial and lateral, the enamel thickens somewhat. In the thick IPI, the IPM is perpendicular to the HSB, the latter decussating at a high angle; the OPI with prism‐parallel IPM is 4 to 5 prisms thick. At the EDJ, a zone measuring about 3 prisms shows thick plate‐like IPM forcing the HSB to decussate at a very low angle. The thin OPI shows prism‐parallel IPM over a thickness of 2 to 3 prisms. The PE has radial enamel. The entire incisor cross section could not be evaluated; the labial part of the enamel is less flat than in *Gregorymys* and *Entoptychus*.

##### Geomyidae: Geomyinae

###### *Geomys* sp. (Figures 2c and 3c), Early Pliocene: early Blancan, KOE 3511

*Geomys* sp. shows a three‐layered schmelzmuster (schmelzmuster type 2a) with mostly transversely but toward mesial also slightly diagonally oriented HSB and steep inclination. The decussation angle of the HSB is high to perpendicular with the exception of an about two prims thick zone at the EDJ where they have a very low decussation angle because of thick, plate‐like IPM. The plate‐like character of the IPM gets pronounced at the turn of the enamel toward lateral. The IPM is at right angles in the IPI and prism‐parallel in the thin, 1–2 prism thick OPI. The PE shows radial enamel. The incisor cross section is triangular‐shaped with labially flat enamel.

###### cf. *Geomys quinni* (Figures 2b and 3b), Early Pliocene: early Blancan, KOE 3248

cf. *Geomys quinni* shows a three‐layered schmelzmuster (schmelzmuster type 2) with transversely oriented HSB throughout the entire enamel; the HSB are steeply inclined. The IPI shows HSB with perpendicular IPM, and the OPI measures only 3–4 prisms and has prism‐parallel IPM. There is a conspicuous, 3–4 prism thick zone at the EDJ consisting of thick, plate‐like IPM allowing HSB to decussate only at a very low angle. The HSB decussation angle gets larger toward the PI/PE junction but seems not to be fully perpendicular. The PE consists of radial enamel. The incisor cross section is triangular‐shaped with labially flat enamel.

###### *Geomys bursarius* (Figure 3d), Holocene, KOE 3275; *Geomys bursarius* (Figure 3e), extant (ca. 600 BP), KOE 3279

The schmelzmuster is three‐layered (schmelzmuster type 2a) with transversely oriented HSB, which transition to a slightly diagonal orientation to the lateral and mesial parts of the enamel. In the IPI, the IPM is perpendicular to the prism long axes. This angle becomes low acute to parallel in the 2–4 prism thick OPI, which is best discernable in the longitudinal section. Also, HSB layers decussate at varying angles: angles closer to the EDJ are about 45 degrees and rise closer to 90 degrees, never being fully perpendicular. There is a conspicuous zone at the EDJ, where the IPM is thick and plate‐like forcing the first four HSB layers to decussate at a very low angle. Toward the lateral parts of the enamel, this zone measures even 6 prism layers. The PE is made up of radial enamel. The enamel surface is wrinkled resulting in a wavy appearance in transverse sections. The incisor cross section is triangular‐shaped with labially flat enamel in KOE 2379; incisor cross section is subtriangular in shape with somewhat more rounded enamel in KOE 3275.

###### *Cratogeomys castanops* (Figures 2e and 3f), Late Pleistocene (ca 20,000 BP), KOE 3284

The schmelzmuster is three‐layered (schmelzmuster type 2), and HSB are oriented transversely throughout the entire enamel; the HSB are steeply inclined. The PI is twofold having an IPI, in which IPM is perpendicular to the prisms and a 1–3 prism thick OPI, in which IPM is at low angle or parallel to the prisms. Right at the EDJ is a four prism thick zone with thick, plate‐like IPM, forcing the decussation angle of the HSB to about zero. Toward the junction PI/PE, the HSB decussation angle rises reaching 90 degrees. The PE consists of radial enamel. The OES is wrinkled causing a wavy OES as seen in transverse sections. The incisor cross section is subtriangular in shape with a rounded dentine body and labially flat enamel.

###### *Pliogeomys buisi* (Figure 3a), Late Miocene/Early Pliocene: Late Hemphillian, KOE 3500

Three‐layered schmelzmuster (schmelzmuster type 2) features steeply inclined, transversely oriented HSB decussating at right angles. The thick IPI shows IPM that is mostly perpendicular to the prisms; at the EDJ, however, is a two prism thick zone with plate‐like IPM inducing a quite reduced angle of HSB decussation. The OPI with prism‐parallel IPM measures only 2 to 3 prisms and is best observable in the longitudinal section. The PE is made up of radial enamel. The incisor cross section is triangular‐shaped with labially flat enamel.

###### *Thomomys bottae* (Figures 2d and 3j), Late Pleistocene (ca 20,000 BP), KOE 3283; *Thomomys talpoides* (Figures 2a and 3k), extant, KOE 650 (figured in Kalthoff, 2000: fig. 40: a1)

Both species of the genus *Thomomys* feature a three‐layered schmelzmuster (schmelzmuster type 2a) with transversely to slightly diagonally oriented HSB with steep inclination. The PI is twofold with a thick IPI with perpendicular IPM and an only 4 prism thick OPI with prism‐parallel IPM. There is a conspicuous zone at the EDJ where the IPM is thickened and plate‐like among the HSB, forcing the latter to decussate at low angle. This zone is especially well expressed in *T. bottae* where it gets even more obvious toward the more lateral and mesial parts of the enamel band in transverse sections. The PE consists of radial enamel that passes into primitive radial enamel in the central part of the enamel band close to the OES. The incisor cross section is triangular‐shaped with labially flat enamel in both *Thomomys* species.

##### Heteromyidae: Mioheteromyinae

###### *Schizodontomys sulcidens* (Figures 3q and 4b), Early Miocene: latest Arikareean, KOE 3270

*Schizodontomys sulcidens* shows a three‐layered schmelzmuster (schmelzmuster type 2). The PI has transversely oriented, steeply inclined HSB with IPM at right angle to the long axes of the prisms in the IPI; this angle is low or IPM is parallel over 2 to 3 prism rows in the OPI. The PE is made up of radial enamel. The incisor cross section is oval‐shaped with rounded enamel.

**FIGURE 4:**
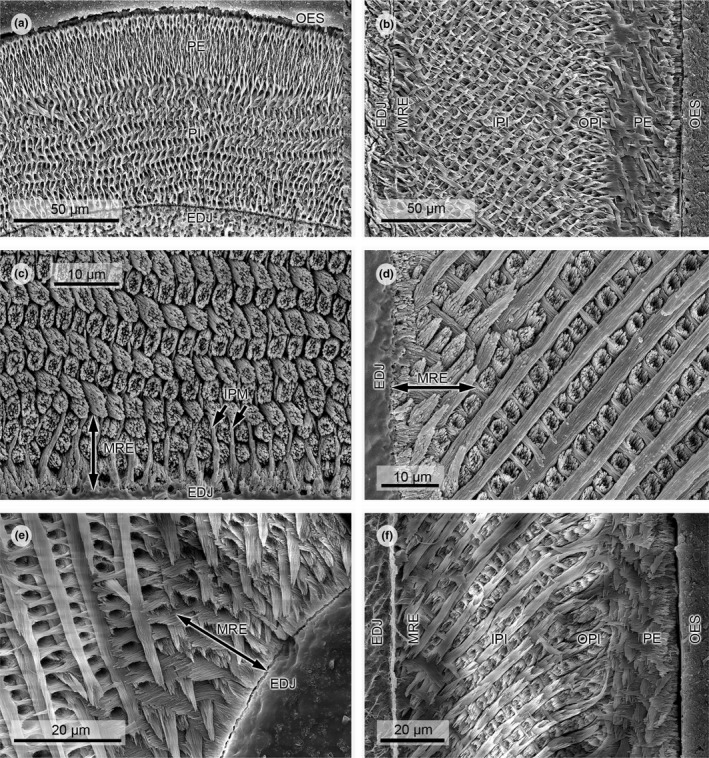
Scanning electron micrographs of lower incisor enamel microstructure in Heteromyidae. (a) *Perognathus* *mclaughlini*, transverse section, KOE 3505. This taxon shows the typical two‐layered schmelzmuster of the perognathine Heteromyidae. (b) *Schizodontomys sulcidens*, longitudinal section, KOE 3270. Modified radial enamel is thin and extends over two to three prism rows. (c and d) *Dipodomys ordii*, KOE 1011. (c) detail of transverse section; (d) detail of longitudinal section. Modified radial enamel is well‐visible both in transverse and in longitudinal sections. (e) *Perognathus* *merriami*, detail of transverse section at bend toward mesial, KOE 3274. The plate‐like IPM is forcing the prisms to run parallel to each other. (f) *Cupidinimus nebraskensis*, longitudinal section, KOE 3259. This Miocene dipodomyine heteromyid shows a three‐layered schmelzmuster with well‐expressed modified radial enamel. Abbreviations: EDJ, enamel–dentine junction; IPI, inner portio interna; IPM, interprismatic matrix; MRE, modified radial enamel; OPI, outer portio interna; OES, outer enamel surface; PE, portio externa; PI, portio interna

##### Heteromyidae: Dipodomyinae

###### *Cupidinimus nebraskensis* (Figures 3m and 4e), Middle Miocene: late Barstovian, KOE 3259; *Cupidinimus* cf. *cuyamensis* (Figure 4b), Middle Miocene: late Barstovian, KOE 3496

The schmelzmuster in both *Cupidinimus* species is three‐layered (schmelzmuster type 2) and has transversely oriented, steeply inclined HSB in the entire enamel. The twofold PI consists of an IPI with perpendicular oriented IPM and a 1–3 prism thick IPI with prism‐parallel IPM. At the EDJ is an about three prism zone with thick, plate‐like IPM, allowing the HSB still to decussate, but only at a low angle. The HSB decussation angle is high in the remaining part of the PI but does not reach 90 degrees. The PE is made up of radial enamel. The incisor cross section is oval‐shaped in both species of *Cupidinimus*, the middle labial part of the enamel is flattened in KOE 3496; this feature cannot be evaluated in KOE 3259.

###### *Dipodomys ordii* (Figures 3n and 4c and d), extant, KOE 1011 (figured in Kalthoff, 2000: fig 41: b1; plate 13, fig 7); *D*. *ordii* (Figure 3o), extant, KOE 1601

The schmelzmuster in both specimens is two‐layered (schmelzmuster type 3) and shows transversely oriented, moderately to steeply inclined HSB throughout the entire enamel. The HSB decussation angle in the PI is high. The IPM is perpendicular to the prism long axes in most of the PI but angle decreases markedly in a 2 to 3 prism thick zone before the junction PI/PE. At the EDJ, the IPM is thickened and plate‐like in a zone of 2 to 3 prism thickness; the HSB still decussate but at low angle (*D. ordii*, Oregon) or are parallel (*D. ordii*, California). The PE consists of radial enamel that in its outermost portion merges into primitive radial enamel. The incisor cross section is oval‐shaped and the middle labial part of the enamel is flattened.

##### Heteromyidae: Heteromyinae

###### *Heteromys anomalus* (Figure 3p), extant, KOE 4231

*Heteromys anomalus* has a two‐layered schmelzmuster with mostly transversely oriented HSB and moderate inclination; mesially HSB become diagonal (schmelzmuster type 3a). The thick PI shows IPM at right angles but the angle is markedly reduced over 2 to 3 prisms at the junction PI/PE. At the EDJ, the IPM is thickened and plate‐like in a zone measuring 3 to 4 prisms. The PE is made up of radial enamel. The incisor cross section is pear‐shaped with rounded enamel.

##### Heteromyidae: Perognathinae

###### *Perognathus mclaughlini* (Figures 3r and 4a), *Perognathus rexroadensis* (Figure 3s), Early Pliocene: early Blancan, KOE 3505, 3507; *Perognathus bibalii* (Figure 3t), Pleistocene/Holocene, KOE 3252; *Perognathus merriami* (Figures 3u and 4e), Holocene, KOE 3274

The schmelzmuster is two‐layered (schmelzmuster type 3) in all four species of *Perognathus*. The HSB are transversely oriented in the entire enamel; the inclination is steep in the Early Pliocene taxa and moderate in the Pleistocene/Holocene taxa. The IPM is perpendicular to the prism long axes in the PI, yet the angle decreases somewhat near the junction PI/PE. In a zone at the EDJ, the IPM is thickened and plate‐like and does not anastomose between the subparallel HSB; this zone is best expressed at the bending of the enamel laterally (*P. rexroadensis*) or medially (*P. mclaughlini*, *P. merriami*), respectively. The PE consists of radial enamel. The incisor cross section is oval‐shaped in all four species of *Perognathus*; the middle labial part of the enamel is somewhat flattened in *P. bibalii* and *P. merriami*.

###### *Chaetodipus penicillatus* (Figure 3v), extant, KOE 1602 (figured in Kalthoff, 2000: fig 41: a1; plate 13, fig 6)

The schmelzmuster is two‐layered (schmelzmuster type 3). The HSB are moderately inclined and transversely oriented in the enamel. The PI is thick and the IPM is perpendicular to the prism long axes; this angle decreases close to the junction PI/PE. At the EDJ is a conspicuous, 2–3 prism thick zone with thick, plate‐like IPM, in which HSB decussate at low angle. The PE is made up of radial enamel and a thin PLEX at the OES. The incisor cross section is oval‐shaped and the middle labial part of the enamel is flattened.

#### 3.1.2 Geomyina

##### Heliscomyidae

###### *Heliscomys* sp. (Figures 3w and 5a), Early Oligocene: Orellan, KOE 3466; *Heliscomys vetus* (Figures 3x and 5b), Late Eocene: Chadronian, KOE 3528

*Heliscomys* sp. and *Heliscomys vetus* have a three‐layered schmelzmuster (schmelzmuster type 2a), in which HSB are oriented transversely to diagonally with a high inclination. The IPM in the IPI is perpendicular to the prisms, this angle decreases markedly in the thin OPI, which measures only 1–2 prisms. The PE consists of radial enamel. The incisor cross section is slender and oval‐shaped and the middle labial part of the enamel is flattened.

**FIGURE 5:**
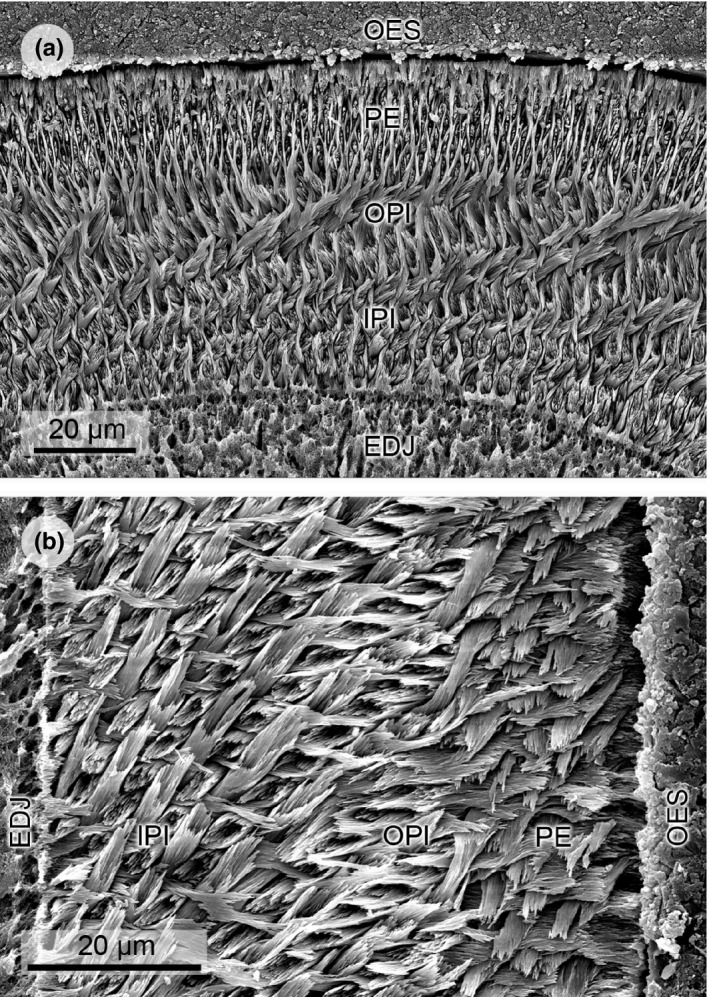
Scanning electron micrographs of lower incisor enamel microstructure in Heliscomyidae. (a) *Heliscomys* sp., transverse section, KOE 3466. The portio interna is two‐layered with perpendicular IPM in the IPI and prism‐parallel IPM in the OPI. (b) *Heliscomys vetus*, longitudinal section, KOE 3528. HSB are highly inclined. Abbreviations: EDJ, enamel–dentine junction; IPI, inner portio interna; IPM, interprismatic matrix; OPI, outer portio interna; OES, outer enamel surface; PE, portio externa

## 4 DISCUSSION

The enamel microstructure is comparatively homogeneous in Geomyidae with respect to schmelzmuster and enamel thickness (Table 1). Entoptychinae (*Pleurolicus, Entoptychus, Gregorymys*) and Geomyinae (*Pliogeomys, Geomys, Cratogeomys, Thomomys*) have a three‐layered schmelzmuster with transversely and, in part, diagonally oriented HSB. The PI is twofold with IPM at different, mostly high angles to the prism long axes in a thick IPI and prism‐parallel IPM in a thin OPI; the PE consists of radial enamel. Enamel is moderate to greatly thick in both Entoptychinae and Geomyinae. From an evolutionary perspective, this schmelzmuster is moderately derived and represents types 2 and 2a of Kalthoff (2000). The incisor cross section is triangular or subtriangular with characteristically flattened enamel in all analyzed taxa (Figure 3a–k).

Compared to Geomyidae, the enamel microstructure in Heteromyidae is almost as homogeneous but is more derived. Perognathinae (*Perognathus, Chaetodipus*), and the extant Dipodomyinae (*Dipodomys*) and Heteromyinae (*Heteromys*) have schmelzmuster type 3 with transversely oriented HSB and angled IPM in an only onefold PI and radial enamel in the PE. The Miocene (Barstovian) dipodomyine *Cupidinimus* and the Miocene (Arikareean) mioheteromyine *Schizodontomys* feature a more plesiomorphic, three‐layered schmelzmuster of type 2, similar to that described for geomyids. The thickness of the enamel varies somewhat from very thick in *Schizodontomys* and *Heteromys* to moderate/thick in perognathines, and from moderate/thick in Miocene dipodomyines to thick in extant dipodomyines. The incisor cross section is oval with a flattened middle labial part of the enamel with the exception of *Schizodontomys*, *Heteromys*, *Perognathus* *rexroadensis*, and *P. mclaughlini*, which all have rounded enamel (Figure 3p–s).

The schmelzmuster types 2 and 3 occurring in Geomyidae and Heteromyidae, respectively, are represented in the lower incisors of various other fossil and extant rodent clades with uniserial HSB: the more uncommon type 2 in members of Deomyinae, Murinae, and Neotominae; the very common type 3 in Arvicolinae, Cricetinae, Dendromurinae, Gerbillinae, Murinae, Myocricetodontinae, Nesomyinae, Otomyinae, Sigmodontinae, and *Trilophomys* (Kalthoff, 2000). Schmelzmuster types 2 and 3 evolved recurrently in rodent incisors and, therefore, are homoplastic structures conveying limited phylogenetic value.

A remarkable structure occurs at the EDJ in both Geomyidae and Heteromyidae: thickened IPM fibers form plates between prism rows in a zone of 3–4 prism thickness; the plate‐like IPM is perpendicular to the prism long axes and does not anastomose. The IPM plates relate to the inter‐row sheets of Boyde (1969); however, the sheets are at most half as thick as a prism, that is, 1–1.5 µm. The inter‐row sheets induce a strong reduction of the HSB decussation angle, forcing them to arrange in nearly or fully parallel orientations. The inter‐row sheets were incorrectly identified as a “starting zone” by Kalthoff (2000).

Combined, these characters (IPM developed as inter‐row sheets, direction of IPM inter‐row sheet fibers perpendicular to prism direction, subparallel prisms in radial rows) define the modified radial enamel of Pfretzschner (1993, 1994). In high‐crowned teeth of large mammals (and in rodent incisors as variety of hypsodont teeth) prisms steeply ascend toward the occlusal surface as a reaction against increased abrasion (Rensberger & Koenigswald, 1980). In addition, special microstructures may occur adjacent to the EDJ as a reaction to tension forces (Pfretzschner, 1993, 1994), all showing decussation of linear elements in a radial–vertical direction. In modified radial enamel, these elements are the (sub)parallel prisms and the interjacent inter‐row sheets.

We detected modified radial enamel close to the EDJ exclusively within Geomyoidea but not in Geomorpha outgroups, such as Heliscomyidae; nor was modified radial enamel reported to occur in the lower incisors of Eomyidae (Wahlert & Koenigswald, 1985) or Florentiamyidae (Wahlert, 1983). As this enamel type was found to be biomechanically beneficial for reducing tension and bending forces in high‐crowned teeth, we assume a similar form‐function association in Geomyidae and Heteromyidae (Pfretzschner, 1993, 1994; Vassallo et al., 2021). Consequently, we explain the presence of modified radial enamel as an adaptation to prevent structural failure triggered by increased mechanical stress acting on this tooth position due to regular burrowing activities (including chisel‐tooth digging) and feeding on abrasive, fiber‐rich plants and plant parts that grow underground (e.g., forbs, roots, stems, bulbs, tubers).

In general, the lower incisors are the more active gnawing teeth in rodents compared to the upper pair, a fact that has been quantified by several studies: A recent experimental analysis on the kinematics of chisel‐tooth digging by African mole rats showed that the upper incisors are used as an anchor while the lower incisor excavates the soil (Van Wassenbergh et al., 2017). Judging from the displacement of the respective incisors during excavation, the work—and with that, concomitant compressive stress—of the lower incisors is three times greater than that of the upper pair (Van Wassenbergh et al., 2017). Large differences in yearly growth rates between upper and lower incisors in the chisel‐tooth digging pocket gophers *Thomomys talpoides* (upper 25 cm/year; lower incisor 45 cm/year; Miller, 1958) and *T. bottae* (upper: 23 cm/year; lower 36 cm/year; Howard & Smith, 1952) corroborate this conclusion. It should be mentioned, however, that we also found modified radial enamel in the upper incisors of, for example, *Thomomys talpoides*, *Chaetodipus penicillatus,* and *Dipodomys ordii*.

All geomyids have a fossorial lifestyle (e.g., Gomes Rodrigues et al., 2016; Reichmann, 2007) meaning that they primarily, but not exclusively, live underground. They are committed diggers. Scratch‐diggers, such as *Geomys* (and probably also *Pliogeomys*; Flynn et al., 2008; Joeckel & Tucker, 2013), rely on their strong and long claws for digging and they use incisors to remove rocks and cut roots (Lessa & Thaeler, 1989). In contrast, chisel‐tooth diggers, such as the markedly proodont *Thomomys,* use their broad, triangular incisors to loosen soil to build their extended burrow systems (Jones & Baxter, 2004). Interestingly, fossoriality was established early in geomyid history as remains of early Oligocene (early Arikareean, Ar1) *Gregorymys* were found inside a burrow system (Jiménez‐Hidalgo et al., 2018; Ortiz‐Caballero et al., 2020). The burrows show incisor marks but lack claw marks, contrary to early Miocene (Arikareean) presumed *Gregorymys* burrows that show a combination of incisor and claw marks (Gobetz & Martin, 2006). However, there is contradictory evidence regarding chisel‐tooth digging in entoptychine gophers: the skull of *Gregorymys* does not display the necessary adaptations for this burrowing mode (Calede et al., 2019) whereas skulls and postcranial material of *Pleurolicus* and *Entoptychus* show specializations for fossoriality and are in that respect most similar to extant tooth‐digging species (Calede et al., 2019; Rensberger, 1971, 1973).

Heteromyids either are terrestrial generalists (*Heteromys*, *Chaetodipus*, *Perognathus*) or demonstrate ricochetal/saltatorial locomotion (*Dipodomys*, *Microdipodops*). All species excavate burrows, which can vary in architecture from simple tubes (*Microdipodops*) to extended systems (*Heteromys*) (Anderson & Gómez‐Laverde, 2008; O'Farrell & Blaustein, 1974). Cranial morphology and perforations, together with narrow, awl‐shaped incisors with an oval or somewhat labially flattened cross section, argue for tunnel construction by scratch‐digging. Ricochetal/saltatorial adaptations were suggested for the middle Miocene (Barstovian) dipodomyinae *Cupidinimus* (Wood, 1935) and for the early Miocene (Arikareean) Mioheteromyinae *Schizodontomys* (Calede et al., 2019; Rensberger, 1973; Voorhies, 1975; Wood, 1935). A reviewer pointed out that the early geomyoid *Tenudomys dakotensis* and some basal heteromyids likely had a terrestrial (*Bursagnathus* *aterosseus*) or even arboreal (*Proheteromys latidens*) lifestyle (Calede et al., 2019).

Geomyids and heteromyids are sister taxa, sharing a common, yet unknown, ancestor. The presence of modified radial enamel close to the EDJ in both clades throughout all taxa suggests acquisition of this character in the ancestor, which we assume had a fossorial lifestyle and employed tooth‐digging as the primary excavation mode. We interpret the presence of modified radial enamel as biomechanically advantageous for geomyids engaged in tooth‐digging and/or using their large, triangular and flattened lower incisors for dirt and root removal during scratch‐digging. Although heteromyids self‐construct burrows, use of their incisors as digging tools or aids has never been reported; moreover their skull architecture and incisor shape argue against such behavior (Calede et al., 2019; McIntosh & Cox, 2016). We regard lower incisor modified radial enamel in Heteromyidae as a retained plesiomorphic character that, apparently, is selectively neutral.

Modified radial enamel might still be biomechanically advantageous for dietary specialists living in desert conditions, such as the heteromyid *Dipodomys microps*. This species has flat, chisel‐shaped lower incisors, with which it peels the salt‐coated outer leaf layers of halophytic plants (Kenagy, 1972) to reach the inner, soft tissues; a behavior that might impose certain stresses on incisors.

Interestingly, modified radial enamel is absent from fossorial rodents in other representatives of the mouse‐related clade, such as Myospalacinae (zokors), Rhizomyinae (bamboo rats, African mole‐rats), Spalacinae (blind mole‐rats), the murine *Nesokia indica* (Short‐tailed Nesokia), and the arvicoline *Ellobius murinus* (Northern Mole Vole) (Ayudhya & Wannaprasert, 2020; Kalthoff, 2000). On the other hand, modified radial enamel also occurs in the lower incisors of a few other muroid taxa with uniserial Hunter‐Schreger bands (Kalthoff, 2000). These are (a) the Congo Forest Rat *Deomys ferrugineus* (Deomyinae), a terrestrial species with no burrowing activities (Ray & Malcolm, 2013); (b) Milne‐Edwards's Tufted‐tail Rat *Eliurus myoxinus* (Nesomyinae), which is a scansorial species dependent on forest environments (Goodman, 2016); (c) Bastard's Big‐footed Mouse *Macrotarsomys bastardi* (Nesomyinae), which has terrestrial adaptations but uses self‐constructed burrows (Carleton & Goodman, 2003); and (d) the Southern African Pouched Mouse *Saccostomus campestris* (Cricetomyinae), showing terrestrial and scansorial adaptations but also short legs and strong toes, well‐adapted to digging (Perrin, 2013). At present, we cannot explain the occurrence and obviously parallel evolution of modified radial enamel in these species. No underlying phylogenetic signal seems to be present and, regarding biomechanics, it remains to be tested whether modified radial enamel can be linked to specialized foraging behavior like, for example, insectivory in *Deomys ferrugineus*.

In the light of the above reported evidence of modified radial enamel in some taxa unrelated to Geomyoidea and among each other, a reviewer suggested that this enamel trait might as well be homoplastic in Geomyidae and Heteromyidae. However, we disagree because modified radial enamel is a character consistently present in both families. This is a clear difference to the above examples, where this character occurs isolated.

South American burrowing rodents with multiserial Hunter‐Schreger bands have been studied by Vieytes et al. (2007). The authors did not mention the occurrence of modified radial enamel but judging from their images of upper incisors, HSB decussation appears to be significantly reduced at the EDJ in *Octodon “bridgesi”* and in *Dactylomys boliviensis* but we could not evaluate whether the IPM is thick and plate‐like (Vieytes et al., 2007: figure 2G, H). A biomechanical reinforcement similar to that in geomyids and heteromyids is possibly present in these taxa.

### 4.1 Comparison with Heliscomyidae

Lower incisors of *Heliscomys* sp. and *Heliscomys vetus* (early Oligocene, Orellan) have transverse to slightly diagonal HSB with a twofold PI with perpendicular IPM in the IPI and prism‐parallel IPM in the OPI; the PE consists of radial enamel (schmelzmuster type 2). The incisors are slender and cross sections are oval‐shaped with a rather flat middle labial part of the enamel (Figure 3w‐x).

This moderately derived schmelzmuster type is common in sciurognathous rodents. It is similar to that of geomyids and stratigraphically older dipodomyines, but less derived as that of perognathines, heteromyines, and stratigraphically younger dipodomyines. However, representatives of Heliscomyidae have not developed the zone of modified radial enamel close to the EDJ, which implies that this form of enamel microstructure is restricted to Geomyoidea. Therefore, Heliscomyidae are not ancestral to Geomyoidea as suggested by Asher et al. (2019), which also implies that a ricochetal locomotion for the ancestor of Geomyoidea does not apply.

## 5 CONCLUSIONS

We described the schmelzmuster and lower incisor morphology in a representative sample of fossil and extant Geomyoidea (Geomyidae, Heteromyidae) and compared the results with those in basal geomorphs (Heliscomyidae) and with those in other, nonrelated rodents. We came to the following conclusions:

1. The lower incisor microstructure of fossil and extant Geomyoidea (Geomyidae, Heteromyidae) is characterized by the moderately derived schmelzmuster type 2 (Entoptychinae, Geomyinae, Miocene Dipodomyinae, Mioheteromyinae) and the more derived schmelzmuster type 3 (Perognathinae, extant Dipodomyinae, Heteromyinae).
2. The lower incisor microstructure of Heliscomyidae is characterized by the moderately derived schmelzmuster type 2.
3. In all fossil and extant Geomyoidea, we detected a zone of modified radial enamel close to the enamel–dentine junction (EDJ).
4. The earliest fossil record of this character dates back to at least the early Oligocene (*Gregorymys,*earliest Arikareean, Ar1).
5. We interpret the presence of modified radial enamel as an adaptation to prevent structural failure under increased reaction forces on this tooth position due to significant burrowing activities (including chisel‐tooth digging), underground feeding, and feeding on abrasive, fiber‐rich plants and plant parts.
6. Within Geomorpha, modified radial enamel is restricted phylogenetically to Geomyoidea, for which it is interpreted as ancestral character.
7. Modified radial enamel does not occur in Heliscomyidae, nor was it reported from Florentiamyidae (Wahlert, 1983) or Eomyidae (Wahlert & Koenigswald, 1985). Enamel microstructure characters argue against a close relationship of these extinct families with Geomyoidea and offer an opportunity to rethink the phylogenetic concept of Geomorpha in future.
8. The shared occurrence of modified radial enamel is yet another argument for the close phylogenetic relationship of Geomyidae and Heteromyidae, here on the dental microstructure level.

## CONFLICT OF INTEREST

The authors have no conflict of interest to declare.

## AUTHOR CONTRIBUTIONS

**Daniela C. Kalthoff:** Conceptualization (equal); Data curation (lead); Formal analysis (equal); Funding acquisition (lead); Investigation (equal); Methodology (equal); Project administration (equal); Validation (equal); Visualization (lead); Writing‐original draft (equal); Writing‐review & editing (equal). **Thomas Mörs:** Conceptualization (equal); Data curation (supporting); Formal analysis (equal); Funding acquisition (supporting); Investigation (equal); Methodology (equal); Project administration (equal); Validation (equal); Visualization (supporting); Writing‐original draft (equal); Writing‐review & editing (equal).

## HUMAN AND ANIMAL RIGHTS

This article does not contain any studies with human participants or animals performed by any of the authors.

We declare that some minor adjustments to brightness or contrast have been made to the scanning electron micrographs. Adjustments apply equally across the entire images.

## Supporting information

Appendix S1

Appendix S2

## Data Availability

Specimen details of the Geomyidae, Heteromyidae, and Heliscomyidae taxa analyzed; Raw measurements, schmelzmuster characters, and lifestyle traits for all analyzed taxa: Dryad https://doi.org/10.5061/dryad.15dv41nwt.
