# Supplementary material for: Biomechanical adaptations for burrowing in the incisor enamel microstructure of Geomyidae and Heteromyidae (Rodentia: Geomyoidea)
Source: Ecol Evol. 2021 Jun 16;11(14):9447–59. doi: 10.1002/ece3.7765 (PMC8293781; doi:10.1002/ece3.7765)
Supplement: Supplementary file 1 — Appendix S1 [file ECE3-11-9447-s001.docx]

| **Species** | **Coll. ID** | **Original collection ID, identification level** | **Locality** | **Stratigraphical Age** |
| --- | --- | --- | --- | --- |
| GEOMYIDAE |  |  |  |  |
| *Thomomys talpoides* | KOE 650 | ZSHD 1397 (I inf. from dentary with molars) | California 1863 | Extant |
| *Geomys bursarius* | KOE 3275 | TMM 41299-12192  (I inf. from left dentary without molars) | Hall’s Cave (Bear Pit,  black dirt) | Holocene |
| *Geomys bursarius* | KOE 3279 | TMM 41455-9, -10  (I inf. from left dentary without molars) | Mac’s Cave | Holocene |
| *Thomomys bottae* | KOE 3283 | TMM 41228-4811, -4812 (I inf. from left dentary with molars) | Dark Canyon Cave  (from lower level) | Late Pleistocene  (ca 20,000 BP) |
| *Cratogeomys castanops* | KOE 3284 | TMM 41228-4813  (I inf. from right dentary without molars) | Dark Canyon Cave  (from lower level) | Late Pleistocene  (ca 20,000 BP) |
| cf. *Geomys quinni* | KOE 3248 | F:AM 87469  (isolated I inf.) | *Stegomastodon*-Quarry, Brown Co., Nebraska | Early Pliocene: early Blancan (Kiem Fm.) |
| *Geomys* sp. | KOE 3511 | UM 28280 (I inf. from dentary with molars) | Loc. UM-K1-47, sec. 35, Fox Canyon, XI Ranch, Meade Co., Kansas | Early Pliocene:  early Blancan |
| *Pliogeomys buisi* | KOE 3500 | UM 60464 (isolated I inf.; compared to I inf. in situ) | Buis Ranch, type area,  Sec. 5, Beaver Co., Oklahoma | Late Miocene - early Pliocene: Late Hemphillian, Hh4 (Ogallala Fm.) |
| *Gregorymys* cf. *curtus* | KOE 3256 | F: AM 105898 (I inf. from dentary with molars) | Royal Valley, Low Marsland Fm.,  lower grey sand | Early Miocene: latest Arikareean, Ar4 (Upper Harrison Beds = "Marsland Fm.") |
| *Entoptychus* sp. | KOE 3244 | AMNH 7134 (I inf. from dentary with molars) | Haystack Valley, Oregon; John Day Fm. | Early Miocene: late Arikareean, Ar3-4 (John Day Fm., Kimberley Mbr) |
| *Pleurolicus* sp. | KOE 3257 | AMNH 7183 (I inf. from left dentary with molars) | John Day Basin,  Oregon | Late Oligocene/Early Miocene: Arikareean |
| HETEROMYIDAE |  |  |  |  |
| *Dipodomys ordii* | KOE 1011 | AMNH 39823 (I inf. from dentary with molars) | Malheur County,  Oregon | Extant |
| *Dipodomys ordii* | KOE 1601 | MVZ RU#36 (I inf. from dentary with molars) | California | Extant |
| *Chaetodipus penicillatus* | KOE 1602 | MVZ (I inf. from dentary with molars) | California | Extant |
| *Heteromys anomalus* | KOE 4231 | NRM 592490 (I inf. from right dentary with molars) | Trinidad and Tobago | Extant |
| *Perognathus merriami* | KOE 3274 | TMM 41299-12191  (I inf. from dentary without molars) | Hall’s Cave (Bear Pit,  black dirt) | Holocene |
| *Perognathus bibalii* | KOE 3252 | F: AM 105902 A (I inf. from left dentary with molars) | Papago Springs Cave, Sonoita, Arizona | Pleistocene/  Holocene |
| *Perognathus mclaughlini* | KOE 3505 | UM 28169 (I inf. from left dentary with molars) | Loc. UM-K1-47, sec. 35, Fox Canyon, XI Ranch, Meade Co., Kansas | Early Pliocene:  early Blancan |
| *Perognathus rexroadensis* | KOE 3507 | UM 28237 (I inf. from left dentary with molars) | Loc. UM-K1-47, sec. 35, Fox Canyon, XI Ranch, Meade Co., Kansas | Early Pliocene:  early Blancan |
| *Cupidinimus nebraskensis* | KOE 3259 | F: AM 105899 (I inf. from left dentary with molars) | Egelhoff Quarry,  Nebraska | Middle Miocene: late Barstovian, Ba2 (Valentine Fm., Cornell Dam Mbr) |
| *Cupidinimus* cf. *cuyamensis* | KOE 3496 | UM 53024 (I inf. from left dentary with molars) | Loc. Norden Bridge Quarry, Brown Co., Nebraska; near base of Valentine Fm. [probably Crookston Mbr according to Wellstead 1981] | Middle Miocene: late Barstovian, Ba2 (Valentine Fm., Cornell Dam Mbr) |
| *Schizodontomys sulcidens* | KOE 3270 | F: AM 105897  (I inf. from right dentary with molars) | Lusk 18 Mile District, Goshen Co., WY | Early Miocene: latest Arikareean, Ar4 (Upper Harrison Beds = "Marsland Fm.") |
| HELISCOMYIDAE |  |  |  |  |
| *Heliscomys* sp. | KOE 3466 | USNM 187565  (I inf. from dentary with molars) | Harrison, N of Munson Ranch, lower part unit 6 | Early Oligocene: Orellan (White River Group: Brule Fm., Orella Mbr) |
| *Heliscomys vetus* | KOE 3528 | UK 68461 (I inf. from dentary with molars) | KU-Nebr.-22, Toadstool Park, Sioux Co., Nebraska, T33N, R53W | Late Eocene: Chadron C, Ch4 (Chadron Fm.) |

Reference

Wellstead, C. F. (1981). Sedimentology of Norden Bridge and Egelhoff fossil quarries (Miocene) of north‐central Nebraska. Transactions of the Nebraska Academy of Sciences, 9, 67–85. https://digitalcommons.unl.edu/tnas/269
