# Supplementary material for: Biomechanical adaptations for burrowing in the incisor enamel microstructure of Geomyidae and Heteromyidae (Rodentia: Geomyoidea)
Source: Ecol Evol. 2021 Jun 16;11(14):9447–59. doi: 10.1002/ece3.7765 (PMC8293781; doi:10.1002/ece3.7765)
Supplement: Supplementary file 2 — Appendix S2 [file ECE3-11-9447-s002.pdf]

Species	Coll. ID	Schmelz muster type	Thickness of E (µm)	PI or IPI (µm)	%	OPI (µm) if present	%	PE (µm)	%	Modified radial enamel	Primary digging mode	Life style	Reference
GEOMYIDAE: Geomyinae													
Thomomys talpoides	KOE 650	2a	140	70	50	15	11	55	39	YES	Chisel-tooth	Fossorial	Verts and Carraway 1999
Geomys bursarius	KOE 3275	2a	116	80	69	10	9	26	22	YES	Scratch	Fossorial	Connior 2011
Geomys bursarius	KOE 3279	2a	110	65	59	15	14	30	27	YES	Scratch	Fossorial	Connior 2011
Thomomys bottae	KOE 3283	2a	94	65	69	7	8	22	23	YES	Chisel-tooth	Fossorial	Jones and Baxter 2004
Cratogeomys castanops	KOE 3284	2	142	96	68	9	6	36	26	YES	Chisel-tooth/Scratch	Fossorial	Davidow-Henry et al. 1989
cf. *Geomys quinni*	KOE 3248	2	91	52	57	12	13	27	30	YES	Scratch	Fossorial	
Geomys sp.	KOE 3511	2a	90	55	61	6	7	29	32	YES	Scratch	Fossorial	
Pliogeomys buisi	KOE 3500	2	97	50	52	16	16	31	32	YES	?Scratch	Fossorial	Flynn 2008; Joeckel and Tucker 2013
GEOMYIDAE: Entoptychinae													
Gregorymys cf. *curtus*	KOE 3256	2	94	43	46	11	12	40	42	YES	Chisel-tooth	Fossorial	Gobetz and Martin 2006; Jiménez-Hidalgo et al. 2018; Calede et al. 2019 (contra chisel-tooth)
Entoptychus sp.	KOE 3244	2a	111	63	57	14	13	33	30	YES	Chisel-tooth/Scratch	Fossorial	Rensberger, 1971, 1973; Calede et al. 2019
Pleurolicus sp.	KOE 3257	2	120	65	54	19	16	36	30	YES	Chisel-tooth/Scratch	Fossorial	Rensberger, 1971, 1973; Calede et al. 2019
HETEROMYIDAE: Dipodomyinae													
Dipodomys ordii	KOE 1011	3	120	85	70	0	0	35	30	YES	?Scratch	Ricochetal, capable of digging complex burrows	Calede et al. 2019
Dipodomys ordii	KOE 1601	3	133	94	71	0	0	39	29	YES	?Scratch	Ricochetal, capable of digging complex burrows	Calede et al. 2019
Cupidinimus nebraskensis	KOE 3259	2	94	59	63	12	13	23	24	YES	??	?Ricochetal/saltatorial	Wood 1935; Voorhies 1975; Calede et al. 2019;
Cupidinimus cf. *cuyamensis*	KOE 3496	2	120	76	63	12	10	32	27	YES	??	?Ricochetal/saltatorial	Calede et al. 2019
HETEROMYIDAE: Heteromyinae													
Heteromys anomalus	KOE 4231	3a	144	116	80	0	0	28	20	YES	?Scratch	Terrestrial generalist; capable of digging extensive burrow systems	Anderson and Gómez-Laverde 2008; Calede et al. 2020
HETEROMYIDAE: Perognathidae													
Chaetodipus penicillatus	KOE 1602	3	110	85	77	0	0	25	23	YES	?Scratch	Generalist; capable of digging burrows, quadrupetal saltation, semiaquatic locomotion	Calede et al. 2019
Perognathus merriami	KOE 3274	3	96	73	76	0	0	23	24	YES	?Scratch	Generalist; capable of digging burrows, quadrupetal saltation, semiaquatic locomotion	Calede et al. 2019
Perognathus bibalii	KOE 3252	3	101	76	75	0	0	25	25	YES	?Scratch	?Generalist	Calede et al. 2019

Perognathus mclaughlini	KOE 3505	3	90	60	67	0	0	30	33	YES	??	?Generalist	Calede et al. 2019
Perognathus rexroadensis	KOE 3507	3	114	75	66	0	0	39	34	YES	??	?Generalist	Calede et al. 2019
HETEROMYIDAE: Mioheteromyinae													
Schizodontomys sulcidens	KOE 3270	2	161	97	60	17	11	47	29	YES	??	Saltatorial, ?fossorial	Wood 1935; Rensberger 1973; Calede et al. 2019
HELISCOMYIDAE													
Heliscomys sp.	KOE 3466	2a	64	33	51	10	16	21	33	NO	??	??	
Heliscomys vetus	KOE 3528	2a	66	35	53	10	15	21	32	NO	??	??	

References

Connior, M. B. (2011). *Geomys bursarius* (Rodentia: Geomyidae). *Mammalian Species* , *43* (879), 104–117. https://doi.org/10.1644/879.1

Davidow-Henry, B. R., Knox Jones, J., & Hollander, R. R. (1989). *Cratogeomys castanops* . *Mammalian Species* , *338* , 16. https://doi.org/10.2307/3504322/2600328

Verts, B. J., & Carraway, L. N. (1999). *Thomomys talpoides* . *Mammalian Species* , *618* , 1–11. https://doi.org/10.2307/3504451/2600769
